# Privacy-aware multi-institutional time-to-event studies

**DOI:** 10.1371/journal.pdig.0000101

**Published:** 2022-09-06

**Authors:** Julian Späth, Julian Matschinske, Frederick K. Kamanu, Sabina A. Murphy, Olga Zolotareva, Mohammad Bakhtiari, Elliott M. Antman, Joseph Loscalzo, Alissa Brauneck, Louisa Schmalhorst, Gabriele Buchholtz, Jan Baumbach

**Affiliations:** 1 Institute for Computational Systems Biology, University of Hamburg, Hamburg, Germany; 2 TIMI Study Group, Division of Cardiovascular Medicine, Department of Medicine, Brigham and Women’s Hospital, Harvard Medical School, Boston, Massachusetts, United States of America; 3 Chair of Proteomics and Bioanalytics, TUM School of Life Sciences, Technical University of Munich, Munich, Germany; 4 Department of Medicine, Brigham and Women’s Hospital, Harvard Medical School, Boston, Massachusetts, United States of America; 5 Faculty of Legal Sciences, University of Hamburg, Hamburg, Germany; University of Southern Denmark, DENMARK

## Abstract

Clinical time-to-event studies are dependent on large sample sizes, often not available at a single institution. However, this is countered by the fact that, particularly in the medical field, individual institutions are often legally unable to share their data, as medical data is subject to strong privacy protection due to its particular sensitivity. But the collection, and especially aggregation into centralized datasets, is also fraught with substantial legal risks and often outright unlawful. Existing solutions using federated learning have already demonstrated considerable potential as an alternative for central data collection. Unfortunately, current approaches are incomplete or not easily applicable in clinical studies owing to the complexity of federated infrastructures. This work presents privacy-aware and federated implementations of the most used time-to-event algorithms (survival curve, cumulative hazard rate, log-rank test, and Cox proportional hazards model) in clinical trials, based on a hybrid approach of federated learning, additive secret sharing, and differential privacy. On several benchmark datasets, we show that all algorithms produce highly similar, or in some cases, even identical results compared to traditional centralized time-to-event algorithms. Furthermore, we were able to reproduce the results of a previous clinical time-to-event study in various federated scenarios. All algorithms are accessible through the intuitive web-app *Partea* (https://partea.zbh.uni-hamburg.de), offering a graphical user interface for clinicians and non-computational researchers without programming knowledge. *Partea* removes the high infrastructural hurdles derived from existing federated learning approaches and removes the complexity of execution. Therefore, it is an easy-to-use alternative to central data collection, reducing bureaucratic efforts but also the legal risks associated with the processing of personal data to a minimum.

## Author summary

Collecting data centrally from different sites in the clinical time-to-event analysis is still challenging due to the high bureaucratic effort and strict data protection laws such as the GDPR. However, huge datasets are needed to extract valuable insights from the data by applying statistical and machine learning approaches. Current approaches are still incomplete: they often do not address privacy issues in any depth, have inaccessible user interfaces, do not cover multiple algorithms, or are not open-source. In contrast, the approach we present in this work is an open-source tool for privacy-aware time-to-event analysis (*Partea*) that can be intuitively used by clinicians, statisticians, and other researchers. It allows the users to run state-of-the-art privacy-aware time-to-event analysis on data distributed between multiple sites through an easy-to-use interface and solves technical and legal issues for the underlying technologies.

## 1. Introduction

Time-to-event analysis is a standard tool in clinical trials to model censored data [1]. In these data, the event of interest (e.g. death or relapse) is not necessarily observed until the end of the study, making usual statistical methods unemployable [2]. Time-to-event analysis is often applied in clinical trials that are designed to identify significant survival-related biomarkers or compare the efficacy of drugs [3–5]. As with many statistical analyses, large sample sizes are needed to produce reliable results and reduce bias. These large sample sizes are usually not available at a single institution. Therefore, different research institutions frequently participate in joint studies using a central data collection strategy. Owing to strict privacy regulations, such as the European General Data Protection Regulation (GDPR), collecting data centrally from different institutions is challenging, imposes substantial bureaucratic burdens, and might even be illegal in some cases [6,7]. Common approaches in clinical data sharing, such as de-identification or anonymization, come with a trade-off between data privacy and data quality [8]. If de-identification is not sufficiently strong, re-identification attacks can still reveal sensitive patient information [9,10]. Successful re-identification of shared anonymized data would harm data subjects in their fundamental right to privacy, thereby exposing the associated researchers to severe legal penalties. This is but one example of how crucial privacy-aware analysis of sensitive biomedical data is for the analysis of clinical studies.

Federated learning (FL) was developed to overcome these obstacles by enabling data analysis on geographically distributed data and keeping the sensitive data private [11,12]. FL allows the training of statistical models without sharing the raw data that contains private information about patients. Only summary statistics or model parameters, so-called local models, are shared with a trusted central aggregator [13]. These local models also fall under GDPR rules if they are generated from personal data. Still, FL systems can add technical security measures to make aggregation possible in a way that would not be the case with the data itself. One fundamental measure is encryption, preventing the aggregation server from being able to mount reconstruction attacks. Moreover, a combination of FL and privacy-enhancing technologies (PETs), such as additive secret sharing or differential privacy (DP), is needed to increase the privacy and security of the whole analysis, reduce the need for trust in the aggregation server, and ensure compliance with data protection laws [14–16]. Such a combination of FL and PETs is often called a hybrid approach. FL or hybrid implementations of various algorithms have already been shown to deliver accurate results in different biomedical applications, such as genome-wide association studies [17,18], differential gene expression analysis [19], the analysis of electronic health records [20], or the prediction of patient outcomes with COVID-19 [21].

For time-time-to-event analysis, the first privacy-preserving and federated approaches were already developed in recent years. A concept of a distributed time-to-event regression was published by Lu et al. in 2015 [22]. *WebDISCO* was a web platform for distributed Cox proportional hazards models without patient-level data sharing. Another approach for calculating federated survival functions using multi-party homomorphic encryption was published by Froelicher et al. in 2021, being the first hybrid approach with an enhanced focus on privacy [23]. The current approaches already show the high potential of FL for time-to-event analysis, however, they do not offer fully extensive solutions. *WebDISCO* is not maintained any longer and does not consider PETs, requiring a high trust in the aggregating server and making it a potential point of cyber-attack [24]. Also, it only supports the Cox proportional hazards model and no other time-to-event algorithms. Froelicher et al. strongly focused on the privacy of the raw data and the exchanged model parameters. However, while their approach offers a strong level of privacy, the resulting survival curves can still leak information about the included patients without much effort [25]. Also, they solely focused on one type of algorithm, the Kaplan-Meier estimator. Another disadvantage is that their tool is unavailable to the general public, and their implementation is not open-source. A comprehensive toolset of widely used time-to-event algorithms is needed that is straightforward to understand and intuitive to set up and use. Ideally, it should reduce technical hurdles to a minimum, achieving similar results to the centralized approaches while preserving the patients’ privacy and being GDPR compliant. Furthermore, when it comes to privacy-aware methods, open-source solutions have tremendous advantages by revealing the source code and therefore increasing the trust in the software. Also, open-source software enables future maintenance, security updates, community-driven development, and code usage in other projects. From a data privacy perspective, the open-source approach has the potential to maintain privacy through faster discovery and remedy of vulnerabilities. At the same time, it poses the risk of hackers exploiting their access to the code. However, from a technical point of view, this risk is not necessarily higher than in closed-source software [26].

To address the existing problems, we propose easily applicable, privacy-aware, federated implementations of the most widely used algorithms in clinical time-to-event studies: survival function, cumulative hazard function, log-rank test, and Cox proportional hazards model. Our implementations are based on a hybrid approach of FL and additive secret sharing to increase the privacy of FL by hiding the shared local statistics and model parameters from the global aggregator [27]. We extended the federated survival function, cumulative hazard function, and log-rank test by a previously published approach to render the resulting outputs differentially private and reduce the privacy leakage of published data [28]. Moreover, we extended the federated Cox proportional hazards model to support L1- and L2 penalization, which was not supported before in *WebDISCO*. We demonstrate that our approach performs as well as centralized approaches. Additionally, we reproduced a multi-institutional clinical study with centralized data collection with very high similarity. All methods are accessible through the open-source platform *Partea* (https://partea.zbh.uni-hamburg.de), enabling complete transparency about the implementations and allowing for further maintenance and extendibility by the community. The platform provides an entire federated infrastructure and makes privacy-aware multi-institutional time-to-event analysis accessible and ready for clinicians, statisticians, and bioinformaticians without deeper technical knowledge. It also incorporates PETs that represent the state-of-the-art in data privacy and ensure sufficient data protection to enable GDPR compliance even in large, complex collaborations. The entire source code is available on GitHub (https://github.com/federated-partea).

## 2. Materials and methods

### 2.1 Implementation

**Fig 1:**
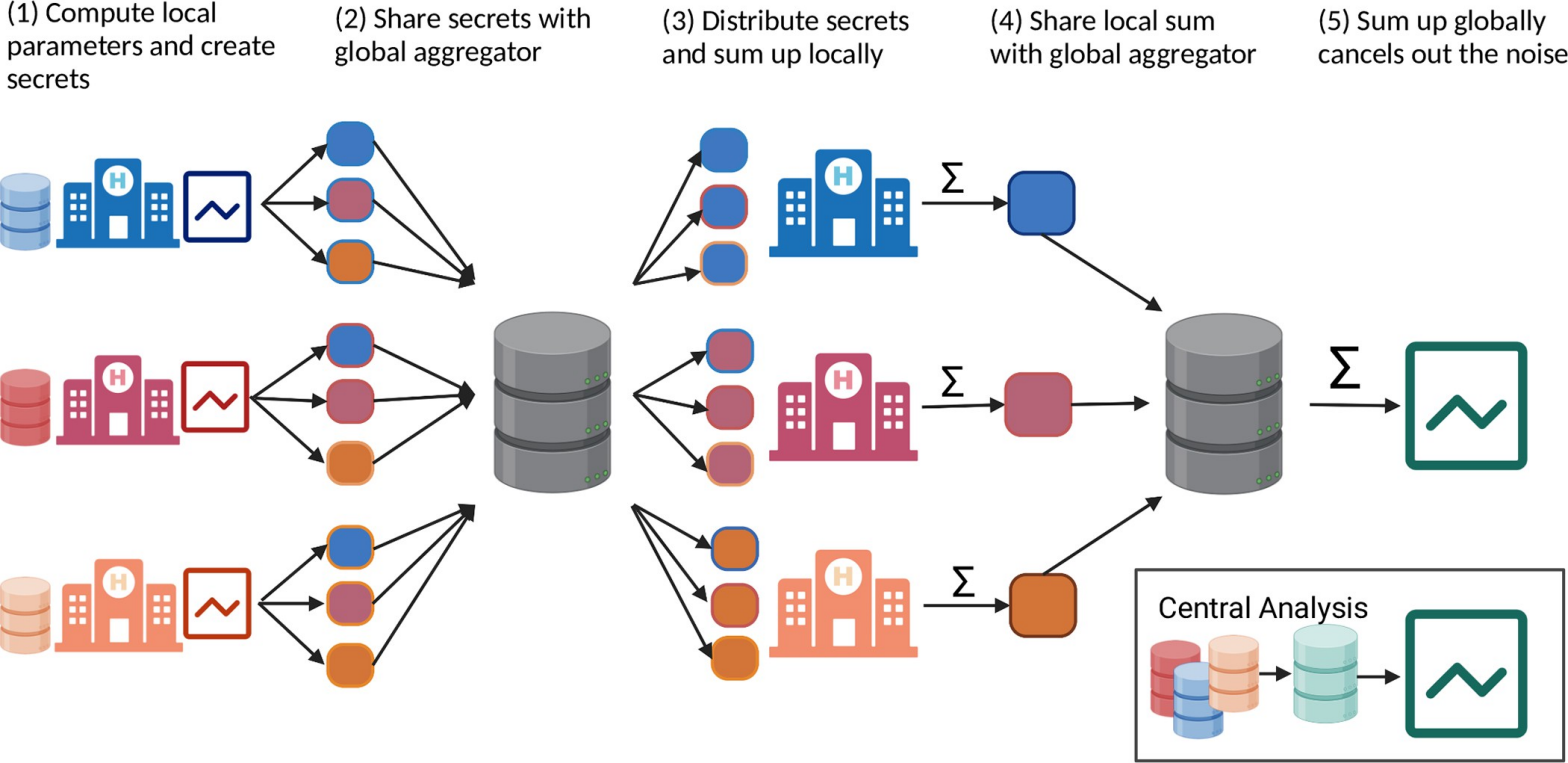
Hybrid federated learning workflow using additive secret sharing. Each institution calculates its local statistics and creates a secret for each participant (1). The global aggregation server receives the secrets and distributes them to the corresponding participants (2). Each local client decrypts the secrets and sums them up (3). The sum is shared with the global aggregation server (4), which sums them up again, revealing the final global aggregation (5). Created with Biorender.com.

In this work, we implemented a hybrid approach combining FL and additive secret sharing to enable privacy-aware multi-institutional time-to-event analysis without central data collection. The FL architecture consists of local clients handling sensitive data analysis at each participating site and a global aggregation server that receives the local parameters from each site to incorporate them into a common, global model. At the beginning of each project, the public keys of each site are exchanged with all other sites. After that, our workflow consists of five major steps, as illustrated in **Fig 1**. (1). Each site creates a secret of its exchanging parameters for each other site, which, summed up together, will reveal the actual parameters again. Each secret is encrypted by the public key of a certain site, and can therefore only be decrypted by this site. (2) The server collects all secrets and distributes them to the corresponding sites. (3) Each site decrypts the received secrets using its private key and sums them up. (4) The summed-up parameters still do not reveal any information and are sent to the aggregation server. (5) Finally, the server sums up the received sums of each site to obtain the actual global aggregate and broadcast it to the local sites. For algorithms with an iterative approach, such as the Cox proportional hazards model, the whole process from step (1) to (5) is repeated until convergence or a stopping criterion has been reached.

The main advantage of this hybrid combination of FL and additive secret sharing is that participants and the aggregating server can only see the global aggregate of the calculation. They are not able to identify or reconstruct any of the exchanged parameters by still maintaining almost identical results. With this approach, we implemented privacy-aware and federated methods for the most commonly used time-to-event algorithms in clinical trials: the Kaplan-Meier estimator for estimating the survival function [29], the Nelson-Aalen estimator for estimating the cumulative hazard function [30], the log-rank test for the comparison of two individual cohorts [31], and the Cox proportional hazards model for time-to-event regression [32,33].

Previous work has shown that it is possible to reconstruct the time of the event and event status directly from the survival function [34,35]. This potential leak in privacy also occurs in survival functions computed on centrally collected datasets. DP can be used to address this potential limitation. In DP, random noise is added to a model to hide the characteristics of individual data points. The noise level is chosen to prevent re-identification but not change the global properties of the dataset [36,37]. Therefore, we integrated the functionality of differentially private survival functions, cumulative hazard function, and log-rank test as proposed by Gondara *et al*. in 2020 into our approach. The authors added random Laplacian noise to the number of events, subjects at risk, and censored individuals for each time point [28].

All algorithms are accessible through the “***Partea***—**P**rivacy-**A**wa**R**e **T**ime-to-**E**vent **A**nalysis” platform, making them easily applicable in clinical trials. *Partea* consists of three main parts: (1) a global web frontend (Angular) to create federated projects, invite participants and visualize the results; (2) a local client application running on all major operating systems (Ubuntu, macOS, Windows) for local computations on sensitive data; (3) and a server for handling the data communication (Django). Through its intuitive user interface, *Partea* is not only applicable to statisticians or (bio)informaticians but can also be used by clinicians or biologists without programming knowledge. After creating a new study and adjusting several initial settings, the study coordinator can invite other participants by sharing unique invitation tokens. With these tokens, an invited participant can join the project through the local *Partea* client, choose its local dataset, and follow the progress of the federated study through the web app. After every participant has joined and the clients are running, the study coordinator can start the federated analysis through the web app. After the run, all results are available through the web app and can be explored interactively or downloaded.

### 2.2 Federated time-to-event analysis algorithms

#### 2.2.1 Survival function, cumulative hazard function, and log-rank test

The survival function S(t) and cumulative hazard function H(t) are defined as:

$$S(t){=\prod }_{t_{i}\leq t}\left[1-\frac{d_{i}}{n_{i}}\right],H(t)=\sum_{t_{i}\leq t}\frac{d_{i}}{n_{i}}$$

In our federated approach, each participating site *k* calculates the number of events *d*_*ik*_ and the number of individuals at risk *n*_*ik*_ locally for each time point *t*_*i*_ and shares the resulting matrix *m*_*k*_ with the global aggregator. The aggregator sums up *d*_*i*_ and *n*_*i*_ of all *K* sites, leading to the formula for the federated survival function, and federated cumulative hazard function *H*_*fed*_(*t*):

$$S_{fed}(t){=\prod }_{t_{i}\leq t}\left[1-(\frac{\sum_{k\leq K}d_{ik}}{\sum_{k\leq K}n_{ik}})\right],H_{fed}(t)=\sum_{t_{i}\leq t}\left(\frac{\sum_{k\leq K}d_{ik}}{\sum_{k\leq K}n_{ik}}\right)$$

Only counts of the observed events and of the individuals at risk are exchanged with the server to calculate the global survival function *S*_*fed*_(*t*) and cumulative hazard function *H*_*fed*_(*t*) by the global aggregator. Using the additive secret sharing scheme for data exchange, the aggregating server can only see the aggregated, global matrix *m* instead of all local matrices *m*_*k*_ that are being received, leading to a similar level of privacy as the centralized approach.

We further extended the approach to allow for the comparison of different cohorts or study groups using the log-rank test. For this comparison, each site needs an additional column in their input data indicating the corresponding group or cohort. For each group c, a separate matrix *m*_*ck*_ is calculated locally and aggregated to a global matrix *m* by the global aggregator. Based on this strategy, a pairwise federated log-rank test statistic $X_{fed}^{2}$ can be calculated centrally at the aggregator using the expected (*E*) and observed (*O*) values of each group pair *A* and *B*:

$$X_{fed}^{2}={\frac{{(O}_{A}-E_{A})}{E_{A}}}^{2}+{\frac{{(O}_{B}-E_{B})}{E_{B}}}^{2}with$$

$$O_{A}=\sum_{i}\left(\sum_{k\leq K}d_{ik}^{A}\right),O_{B}=\sum_{i}\left(\sum_{k\leq K}d_{ik}^{B}\right)\;and$$

$$E_{A}=\sum_{i}\frac{\sum_{k\leq K}n_{ik}^{A}*d_{ik}}{\sum_{k\leq K}n_{ik}},E_{B}=\sum_{i}\frac{\sum_{k\leq K}n_{ik}^{B}*d_{ik}}{\sum_{k\leq K}n_{ik}},$$

#### 2.2.2 Federated Cox proportional hazards model

Further, we reimplemented the WebDISCO [22] approach for the Cox proportional hazards model to enable federated time-to-event regression and extended it by the additive secret sharing scheme. Our implementation is based on lifelines, an open-source, state-of-the-art Python package for time-to-event analysis [38].

As *WebDISCO* did not address any normalization, we extended the approach with a federated z-score normalization. For this purpose, two exchanges with the server are needed. The local mean *m*_*k*_ and the local number of samples *n*_*k*_ for each site *k* and covariate are calculated and shared with the global aggregator to calculate the global mean *m* and share it with the local sites. Thereafter, each site uses the global mean *m* to compute their local ∑_*i*_‖*X*_*i*_−*m*‖^2^ and shares it with the global aggregator to calculate the global standard deviation. This result is broadcast to the local sites again and used to normalize their local data, resulting in the formula for federated z-score normalization:

$${X_{fed,norm}}_{k}=\frac{X_{k}-m_{fed}}{\sigma_{fed}},m_{fed}=\frac{\sum_{k\leq K}m_{k}*n_{k}}{\sum_{k\leq K}n_{k}},\sigma_{fed}=\sqrt{\left(\frac{\sum_{k\leq K}\sum_{i}{\left\|X_{ik}-m_{fed}\right\|}^{2}}{(\sum_{k\leq K}n_{k})-1}\right)}$$

We perform the initialization similar to as was done in the *WebDISCO* approach. After normalization, each site initializes the model statistics based on its local data. These statistics remain the same for the entire training process and are aggregated to the initialized global statistics on the aggregation server.

- *D*_*k*_: distinct event times of site *k*
- *d*_*k*_: number of events at each time point *i* of site *k*
- *z*_*k*_: sum of the covariates over all individuals with an event that occurred at site *k*

resulting in the global aggregates:

$${D=U}_{k\leq K}D_{k},d=\sum_{k\leq K}d_{k},z=\sum_{k\leq K}z_{k}$$

Furthermore, the beta vector containing the coefficient values is initialized with zeros. In our hybrid approach, instead of sharing the distinct event times of each site *k* we share a common, predefined timeline. This hides the actual distinct event times of each site from the global aggregator and assures higher privacy.

Iteratively, until convergence, the global beta vector is broadcasted to the clients and the local statistics are calculated and shared again with the global aggregator:

$$\sum_{l\in R_{i}}\exp\left(\beta^{T}z^{l}\right),\sum_{l\in R_{i}}z_{q}^{l}*\exp\left(\beta^{T}z^{l}\right),\sum_{l\in R_{i}}z_{r}^{l}*z_{q}^{l}*\exp\left(\beta^{T}z^{l}\right)$$

with *R*_*i*_ being the index set of individuals who are at risk for failure at the time *i*. The global aggregator then calculates the first and second-order derivates of the likelihood function, updates the beta vector according to the Newton-Raphson method, and if convergence is not achieved, a new iteration starts.

We further extended the *WebDISCO* approach, to make use of *lifelines* penalized regression functionality and allow the use of both L1 and L2 penalties by specifying the l1-ratio (*α*) and penalty (*p*):

$$\frac{1}{2}p\left((1-\alpha )*{\left\|\beta \right\|}_{2}^{2}+\alpha *{\left\|\beta \right\|}_{1}\right)$$

After convergence, the final coefficients of the model are known and can be used to prepare the final plots and statistics, such as p-values and hazard ratios for each covariate.

## 3. Results

### 3.1 Benchmark evaluation

To evaluate the performance of our approach, we ran analyses on four benchmark datasets that are commonly used in time-to-event analysis: US Veterans’ Administration lung cancer study data [39] (Veteran, 137 samples), NCCTG lung cancer data [40] (Lung, 168 samples), criminal recidivism data [41] (Rossi, 432 samples), and chemotherapy for Stage B/C colon cancer trial data [42] (Colon, 888 samples). More details about the datasets can be found in the supporting information, **S1 Text.** Each dataset was split randomly and equally into 3, 5, and 10 parts to simulate various federated scenarios with different numbers of sites and sample sizes. For this, we simulated a federated environment using docker. Each site’s local client was executed in a separate docker container to simulate network communication and different environments of the local datasets realistically.

#### 3.1.1 Survival function

**Fig 2:**
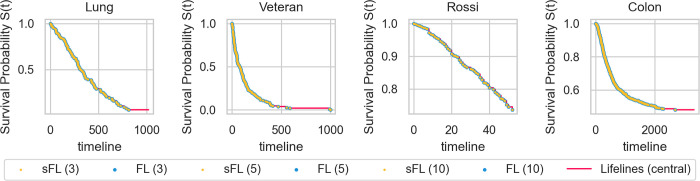
Evaluation of the survival function on benchmark datasets. For both the hybrid approach of FL and additive secret sharing (sFL, yellow) and the federated-only approach (FL, blue), identical survival functions are achieved compared to *lifelines’* Kaplan-Meier estimator (lifelines, red) for all four datasets and the various number of participants.

We calculated the survival function for each federated scenario using the federated approach (FL) and the hybrid approach of FL and additive secret sharing (sFL). We compared it to the central survival function estimated using *lifelines*, a state-of-the-art Python package for time-to-event analysis [38]. Both the federated and hybrid approaches resulted in identical survival functions compared to the central analysis (*lifelines*) for all evaluated datasets and scenarios of varying numbers of participants. The resulting survival curves are shown in **Fig 2**. Owing to the same underlying statistics, this also proves that our FL and sFL approach of the Nelson-Aalen estimator and the log-rank test provide identical results compared to the central analysis.

#### 3.1.2 Differentially private survival functions

We also included the functionality for differentially private survival functions and evaluated the approach by comparing DP survival functions to the actual non-DP survival functions. The main goal of this evaluation was to suggest the privacy loss metric epsilon of the DP computation for future time-to-event analyses. Note that this evaluation is independent of the federated computation, as both provide identical results. In the method for differentially private survival function estimation by Gondara et al. in 2020, they show that the sensitivity of the survival function estimation is 1. With this sensitivity and a variable privacy loss metric epsilon, the amount of noise is calculated, which is added to the survival function to guarantee a certain amount of privacy.

The smaller the privacy loss metric epsilon is, the more privacy is assured by the algorithm. For each dataset, we ran 1000 simulations with different epsilons (3, 2, 1, and 0.75). We next compared each differentially private function to the original non-DP function by applying a log-rank test to test whether two functions significantly differ from each other. A significant log-rank test means that the DP function is not similar to the actual function, making it inaccurate in clinical studies. The results of the log-rank tests are depicted in the supporting information, **S1 Table**.

**Fig 3:**
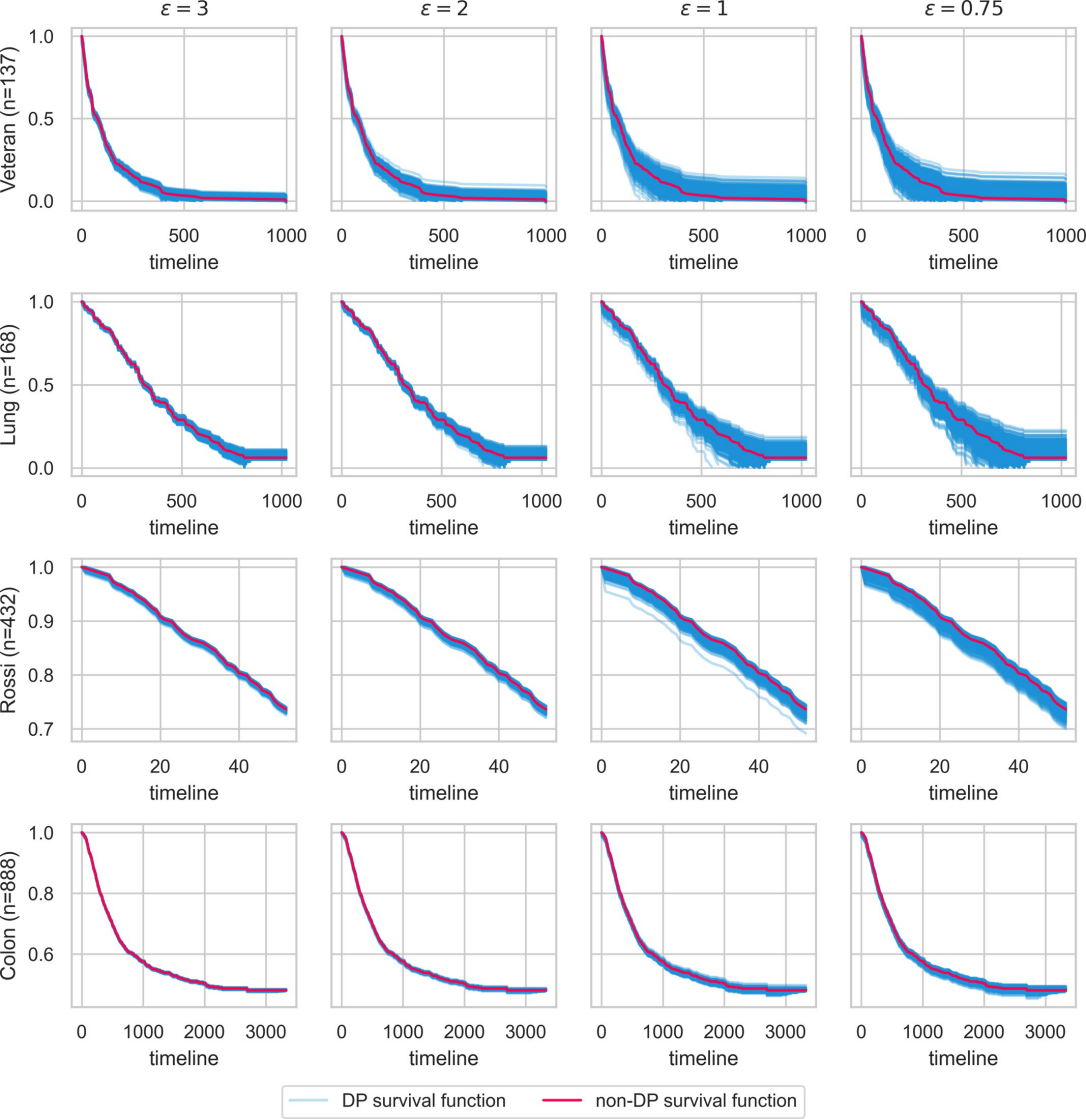
Comparison of DP survival functions against the non-DP baseline. The non-DP survival function (red) is used as a baseline against 1000 runs of DP survival functions for different epsilons and datasets. The resulting DP survival functions (blue) become noisier with decreasing epsilon. Note that the influence of the noise increases with decreasing sample size.

As shown in **Fig 3**, the smaller epsilon is, the greater the resulting survival function differs from the original non-DP survival function. This finding, and the fact that smaller sample sizes are more affected by noise, match the observations in the original publication by Gondara et al. and is a common observation when applying DP.

Using epsilons of 3 and 2 resulted in 100% non-significant differences between the differentially private and non-differentially private survival function, using the log-rank test for all datasets. Except for the two datasets with smaller sample sizes (Lung and Veteran), epsilon equal to 1 and 0.75 led to significantly different survival functions in very few cases (worst being Veteran with an epsilon of 0.75, resulting in 2.4% significantly different functions). This observation indicates that, only in some rare cases, an epsilon of 1 and smaller can lead to too much noise if the sample sizes are small. Again, our results of the DP survival function evaluation are transferable to the Nelson-Aalen estimator and log-rank test as they are all calculated using the same underlying statistics.

Based on this analysis, we suggest three predefined epsilons to reduce complexity and understandability for users: “high DP” with an epsilon of 0.75, which can be applied if more than 400 samples are available; “medium DP” with an epsilon of 1, which can be applied with more than 200 samples; for smaller sample sizes, “low DP” for which an epsilon of 3 should be used.

#### 3.1.3 Cox proportional hazards model

**Fig 4:**
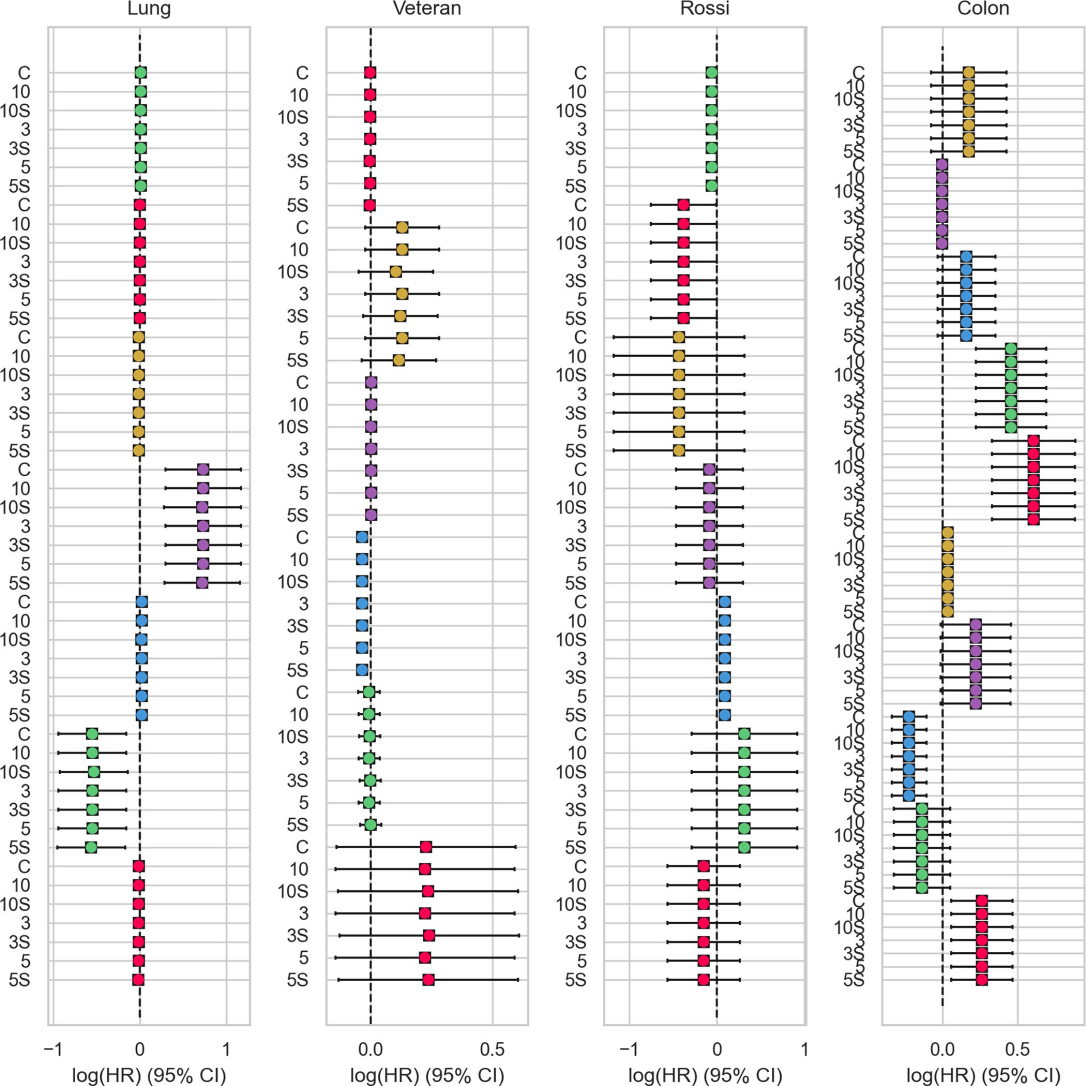
Evaluation of the Cox proportional hazards model on benchmark datasets. For each dataset, we compared the logarithmized hazard ratio and corresponding 95% CI of our algorithms for 3, 5, and 10 clients with the results of the centralized *lifelines* model. For all covariates (distinguished by colors), the federated-only (3, 5, 10) and hybrid approach (S3, S5, S10) resulted in almost identical results compared to the centralized calculation using *lifelines* (C).

Similarly to the evaluation of the survival function, we simulated a federated scenario using the Cox proportional hazards model to compare the resulting logarithmized hazard ratio (HR) and its 95% confidence interval (CI). Fall datasets and the various number of participants, our federated-only approach, and the hybrid approach resulted in almost identical hazard ratios and corresponding CI for all covariates. A detailed overview of the comparison for each covariate and dataset is shown in **Fig 4**.

The evaluation shows that the federated-only approach is identical to the centralized Cox proportional hazards model. The hybrid approach with additive secret sharing is slightly more inaccurate because we not only transmit the timeline of the actual samples. Instead, a time range is used, including intermediate time points not existing in the local datasets. This assures more privacy, as what timepoints are derived from which site is inapparent in the data exchange. As we show on the four benchmark datasets, this slight inaccurateness does not influence the overall interpretation of the results, which remain close to the centralized or federated-only results.

### 3.2 Reproduction of a clinical study

To show the practical benefit of our framework for actual clinical time-to-event studies, we attempted to reproduce the results of the ENGAGE-TIMI 48 study conducted by the TIMI study group [43]. The study data were collected by the TIMI study group as part of a phase three, randomized, double-blind, double-dummy, parallel-group, multi-center, multi-national study, ENGAGE-TIMI 48, and contains more than 21,000 participants [43] from initially more than 1,300 sites. ENGAGE-TIMI 48 compared two different doses of edoxaban, a direct oral factor Xa inhibitor, with warfarin to evaluate the long-term efficacy and safety in patients with atrial fibrillation. Analyses were performed using a Cox proportional hazards model comparing each edoxaban dose group to warfarin and included the two randomization stratification factors. For our analysis, we split the centralized dataset equally into 3, 5, and 10 sites. We used the federated-only and hybrid Cox proportional hazards model to reproduce the results for the five outcome variables: stroke or systemic embolic event (Stroke/see), stroke, see or death from cardiovascular causes (Cv death/stroke/see), major adverse cardiac event (Mace), Stroke, and All-cause death.

**Fig 5:**
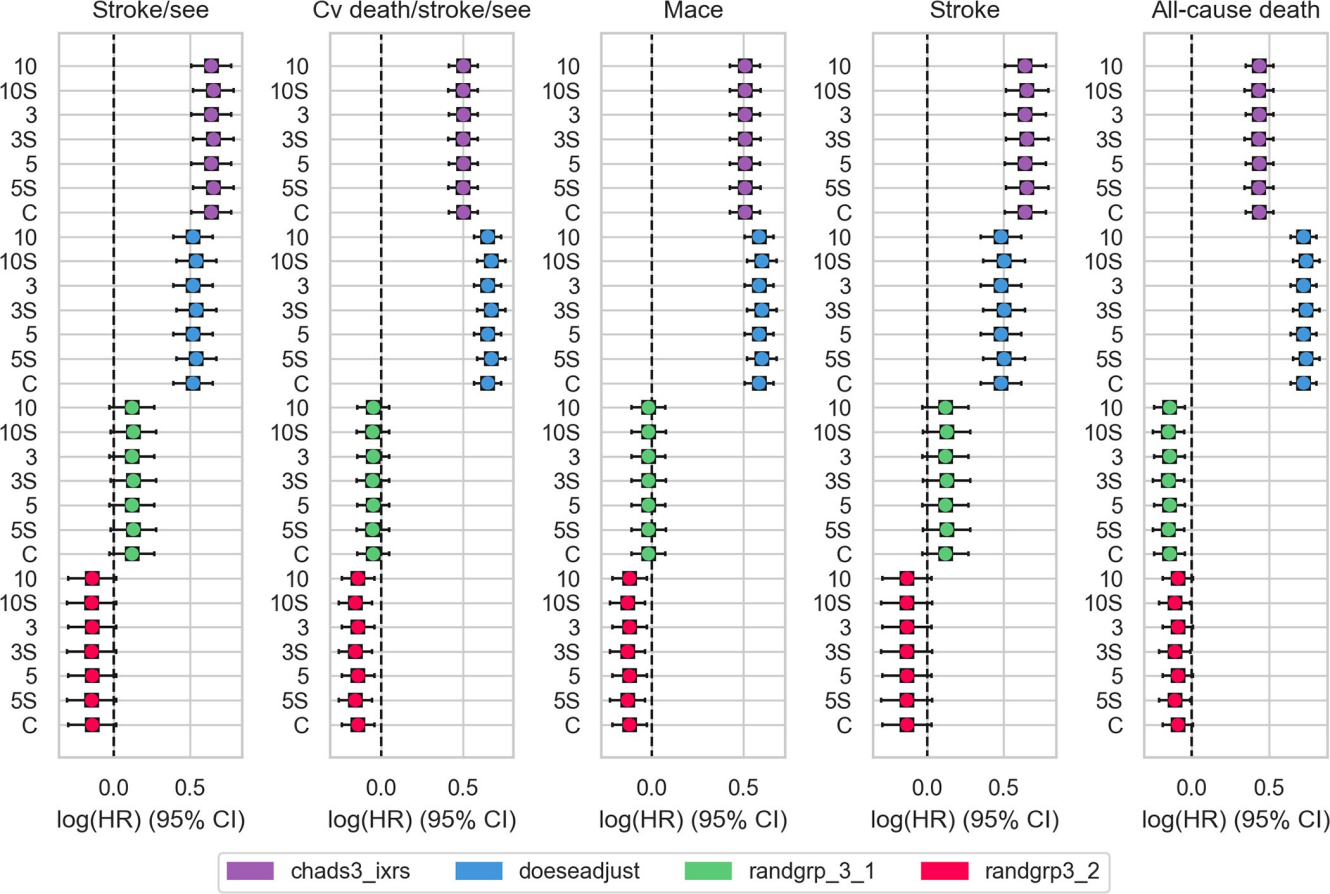
Federated time-to-event analysis of the ENGAGE-TIMI 48 study. Reproducing the ENGAGE-TIMI 48 study using 3, 5, and 10 clients (number) of both federated-only and hybrid approaches (number + S) compared with the results of the central analysis (C). The different covariates (colors) were regressed against five outcome variables (each subplot). The results of all five outcome variables and covariates are highly similar to the central analysis performed using the *lifelines* package.

**Fig 5** depicts the logarithmized hazard ratios for each of the five outcome variables (columns) and covariates (indicated by different colors), calculated with our federated only (3, 5, 10) and hybrid Cox proportional hazards model (3S, 5S, 10S). As apparent in the plots, our results are highly similar to the centralized calculation (C). The number of sites over which the data was distributed does not play any role. This shows that the federated, as well as the hybrid Cox proportional hazard model could accurately reproduce the analysis of the ENGAGE-TIMI 48 study, indicating the high potential of our approaches for future multi-institutional time-to-event studies.

## 4. Discussion

Clinical time-to-event studies are mainly performed on centralized data from one or more institutions. If multiple institutions participate in one study, a complicated data collection strategy is needed with high bureaucratic hurdles and legal pitfalls regarding the privacy of the utilized data. Prior work has already shown the potential of privacy-aware distributed analysis techniques in time-to-event analysis. However, current approaches are not complete. They either are not accessible, only support one kind of algorithm, do not integrate PETs, or are not open-source. In this work, we introduced a hybrid approach of FL and additive secret sharing for the most widely used time-to-event analysis algorithms: the Kaplan-Meier estimator for survival functions, the Nelson-Aalen estimator for cumulative hazard functions, the log-rank test, and the Cox proportional hazards model. All algorithms are bundled in our open-source platform *Partea*, making them easily accessible for usage in clinical trials and increasing trust and maintenance by having a published code-base. Our analyses on several benchmark datasets and the reproduction of a previous clinical study show highly similar results compared to central time-to-event studies. Our platform *Partea* has the possibility of being an intuitive and privacy-aware alternative to central data collection for future multi-institutional time-to-event studies with geographically distributed datasets.

The hybrid approach of FL and additive secret sharing can currently be considered state-of-the-art, privacy-aware, and potentially GDPR compliant. However, evaluating the GDPR compliance of machine learning systems is not trivial, owing to unclear criteria and definitions and the lack of jurisprudence. Even though the status of local and global models as personal data is still uncertain, it is very likely that the GDPR at least remains applicable to local models trained on personal data [15,44]. Likewise, the extent to which the addition of PETs such as additive secret sharing and DP is sufficient to result in GDPR compliance is not conclusively resolved. These questions will need answers from the courts or legislators in the future. The flexibility of *Partea’s* open-source architecture allows it to rapidly be adapted and extended with community input in response to regulatory changes.

Open-source systems like *Partea* have further benefits compared to closed-source systems regarding maintainability and transparency. The source code is openly visible, so everyone can see how personal data is processed, increasing users’ trust. This is also relevant for controllers of personal data, who are legally obligated under Article 32 of the GDPR to protect this data according to the state of art in technical and organizational measures. In the case of open source, the controller can show which PETs are used, how the program treats the data, what is sent around, and whether it holds its promises. In case of any security or privacy issues, users have a much higher chance of discovering this (legally relevant) breach and of holding the data controller accountable. Another advantage is that security gaps can be identified more quickly by the community. One downside of open-source is that it may also facilitate the identification of the existing security breaches for the attackers. However, there are indications that this does not lead to more attacks. In fact, experience has also shown that the security through obscurity of closed-source systems is very brittle [26].

In addition to these promising results, a few problems need to be considered, both technical and legal. Our hybrid scheme is only applicable if at least three sites participate in the analysis. In addition, it is apparent that the results of the hybrid approach differ slightly more from the centralized and federated-only analysis, mainly owing to the stronger privacy mechanism we implemented in this approach. Instead of sharing the exact distinct event times occurring at each site, we use a predefined timeline, including times that do not appear in the local dataset. This approach prevents the global aggregator from reading the event times of a single site. However, users can still use the federated-only approach if they trust the global aggregation server or work with non-sensitive data.

Another problem appearing in most of the federated learning tools is data harmonization. Our algorithms include several preprocessing steps for the computation (e.g. standardization of the data in the Cox proportional hazards model). Also, we allow for a detailed description of the data, such as the used time format (days, weeks, months, years) or the naming of the time and event columns. However, especially in the case of the Cox proportional hazards model, *Partea* expects similar datasets, meaning that besides the event and time columns, all other columns should be harmonized between different sites. The automatic harmonization of data is not trivial and out of the scope of *Partea*. However, this also encourages the participating sites to communicate and discuss a thorough study design upfront which might be even advantageous over an automatic data harmonization.

Our approaches can be easily extended in future work. As already mentioned, by offering an open-source platform and algorithms, Partea can be quickly adapted to potential changes in privacy regulations. Also, subsequent analyses such as checking the proportional hazard assumption based on scaled Schoenfeld residuals could be integrated in the future [45,46]. Furthermore, our platform could be easily extended by further privacy-aware time-to-event analysis implementations, such as Random Survival Forests [47] or Survival Support Vector Machines [48,49]. We believe that through the combination of extendibility through its open-source code, the strong focus on privacy, its accessibility, and its support of the most used time-to-event analysis algorithms, *Partea* has the potential to become the new gold standard in multi-institutional time-to-event analyses and provides various advantages to current solutions.

## Supporting information

S1 Text Dataset. (DOCX)

S1 Table Log-rank test comparison of DP survival functions and non-DP survival functions. For all datasets, using an epsilon of 3 and 2 resulted in non-significant p-values of the log rank-test, indicating that the DP survival functions are still relatable to the original one. Only for the small sample size datasets Veteran and Lung, small epsilons of 1 and 0.75 resulted in significant differences between in less than 2.5% of the curves.
(DOCX)
