# Supplementary material for: Privacy-aware multi-institutional time-to-event studies
Source: PLOS Digit Health. 2022 Sep 6;1(9):e0000101. doi: 10.1371/journal.pdig.0000101 (PMC9931301; doi:10.1371/journal.pdig.0000101)
Supplement: S1 Text — (DOCX) [file pdig.0000101.s001.docx]

# S1 Text

1. The Veterans’ Administration Lung Cancer study data [(Cohen et al. 1982)](https://paperpile.com/c/IdQECu/NhU5) (**Veteran**) was obtained in a randomized trial of two treatment regimens for lung cancer. The dataset contains 137 patients and their corresponding survival time and censoring status. Additionally, the Karnofsky performance score, the months from diagnosis to randomization, the age, and a boolean for prior therapy are included as covariates. The dataset was obtained from the R “survival” package.
2. The NCCTG Lung Cancer Data [(Loprinzi et al. 1994)](https://paperpile.com/c/IdQECu/mYeN) (**Lung**) contains data from 168 patients participating in a study with advanced lung cancer. Besides the survival time, censoring status, age, and sex, it contains various performance scores (ECOG, Karnofsky rated by physician, Karnofsky rated by patient), calories consumed, and the weight loss in the last six months.  The dataset was obtained from the R “survival” package.
3. The Criminal Recidivism Data [(Rossi et al. 1980)](https://paperpile.com/c/IdQECu/eddG) (**Rossi**) contains data of 432 convicts released from Maryland state prisons followed up for one year after release. Half of them were given financial aid; the other half were not. It contains the week of the first arrest after release or censoring and the censoring status (arrested/not arrested). Additionally to the financial aid covariate, age, race, marital status at the time of release, releasement on parole, number of prior convictions, and full-time work experience before incarceration are included.  The dataset was obtained from the R “survival” package.
4. The Chemotherapy for Stage B/C colon cancer trial data [(Laurie et al. 1989)](https://paperpile.com/c/IdQECu/pGaY) (**Colon**) was collected in one of the first successful trials of adjuvant chemotherapy for colon cancer. The dataset was obtained from the R “survival” package. In addition to the time until event or censoring and the censoring status, it contains the treatment type, sex, age, obstruction of the colon by tumor, perforation of the colon, adherence to nearby organs, number of lymph nodes with detectable cancer, differentiation of tumor, extent of local spread, time from surgery to registration, the event type (recurrence or death), and whether there are more than four positive lymph nodes. The dataset was obtained from the R “survival” package.
