# Supplementary material for: Privacy-aware multi-institutional time-to-event studies
Source: PLOS Digit Health. 2022 Sep 6;1(9):e0000101. doi: 10.1371/journal.pdig.0000101 (PMC9931301; doi:10.1371/journal.pdig.0000101)
Supplement: S1 Table — For all datasets, using an epsilon of 3 and 2 resulted in non-significant p-values of the log rank-test, indicating that the DP survival functions are still relatable to the original one. Only for the small sample size datasets Veteran and Lung, small epsilons of 1 and 0.75 resulted in significant differences between in less than 2.5% of the curves. (DOCX) [file pdig.0000101.s002.docx]

# S1 Table

**S1 Table.** **Log-rank test comparison of DP survival functions and non-DP survival functions.** For all datasets, using an epsilon of 3 and 2 resulted in non-significant p-values of the log-rank test, indicating that the DP survival functions are still relatable to the original one. Only for the small sample size datasets Veteran and Lung, small epsilons of 1 and 0.75 resulted in significant differences between in less than 2.5% of the curves.

|  | Veteran (n=137) | Lung (n=168) | Rossi (n=432) | Colon (n=888) |
| --- | --- | --- | --- | --- |
| $\epsilon=3$ | 100% | 100% | 100% | 100% |
| $\epsilon=2$ | 100% | 100% | 100% | 100% |
| $\epsilon=1$ | 99.0% | 99.7% | 100% | 100% |
| $\epsilon=0.75$ | 97.6% | 99.2% | 100% | 100% |
